# Selecting the right method for hypospadias repair to achieve optimal results for the primary situation

**DOI:** 10.1186/s40064-016-3314-y

**Published:** 2016-09-21

**Authors:** Chen Shuzhu, Wu Min, Liu Yidong, Ye Weijing

**Affiliations:** 1 Department of Urology, Ren Ji Hospital, School of Medicine, Shanghai Jiao Tong University, Shanghai, 200127 China; 2 Shanghai Institute of Andrology, Shanghai, 200127 China

**Keywords:** Hypospadias, Male urologic surgical procedures, Treatment outcome

## Abstract

**Background:**

Over the past two decades, Snodgrass tubularized incised plate (TIP) urethroplasty has become one of the dominant surgical techniques with wide applications and excellent cosmetic results. However, TIP has many limitations. We performed a retrospective study at our department and assessed the outcome of the inlay internal preputial graft for extending the applications of TIP.

**Methods:**

Between January 2009 and December 2013, we performed a retrospective study consisting of approximately 508 primary distal and moderate cases. Patients with primary distal hypospadias who had mild or no chordee and good penile development were divided into the following 3 groups based on their procedures: (1) classic TIP hypospadias repair group (n = 198); (2) inlay buccal mucosa graft group (n = 150); and (3) inlay internal preputial graft group (n = 160). The median age was 1.6 years (range 1–4 years). Our data were analyzed statistically by the Chi square test with *P* < 0.05 indicating significant differences.

**Results:**

The mean follow-up period was 18 months (range 6‒24 months). In the classic TIP group, the incidence of urinary fistula and meatal stenosis were both 3.0 % (6/198); in the inlay buccal mucosal graft group, the incidence of urinary fistula was 3.3 % (5/150), and the incidence of stenosis was 2.7 % (4/150); and in the inlay internal preputial graft group, the incidence of urinary fistula was 3.1 % (5/160), and the incidence of meatal stenosis was 4.4 % (7/160). The success rates of each group were as follows: the classic TIP group has a success rate of 93.9 % (186/198); the inlay buccal mucosa graft group had a success rate of 94.0 % (141/150); and the inlay internal preputial graft group had a success rate of 92.5 % (148/160). There were no statistically significant differences between the 3 groups with respect to complication rates.

**Conclusions:**

As the inner foreskin Snodgraft does not appear to be worse than the buccal mucosa graft, it is a good method for hypospadias repair, and this method is not inferior to TIP.

## Background

In 1994, Snodgrass (1994) first reported the use of a tubularized incised plate (TIP) to repair distal hypospadias. During the subsequent 20 years, TIP has become mainstream internationally as the optimal surgical option; however, this operation has higher requirements of the urethral plate, including a wide plate and good quality of the urethra. Although TIP is applicable to many fields, the limitations of TIP surgery have also been gradually revealed. Some scholars have reported that the success rate was significantly lower in crippled cases and in cases with longer neourethras who have received classic TIP. The success rate was shown to decrease in patients who had longer neourethras or multiple previous interventions (Adayener and Akyol 2006). The uroflow curves and fistula positions in patients undergoing TIP repair suggest that the neourethra distal to the fistula may be relatively narrow, creating flow resistance and leading to a proximal fistula; these patients often need regular urethral expansion due to urethral opening or urethral stenosis. A urethral plate incision enables a urethral tubularization, but due to the different features of the urethra, such as narrow urethral plate, shallow urethral groove, or a urethral dorsal incision without epithelium, scar tissue may form (Braga et al. 2007). A longer and newer urethral length means more risk of scar tissue formation, which can create flow resistance and lead to proximal fistula and limitations in urine flow (Perera et al. 2012). Asanuma et al. (2007) summarized several reports about the complications that can occur after TIP urethroplasty, and the mean overall complication rate was 10.8 %, with a 5.7 % incidence of fistula and a 4.7 % incidence of meatal stenosis.

Therefore, some scholars advocate the Snodgraft procedure (Silay et al. 2012), which in our study, includes buccal mucosal (Djordjevic et al. 2011) and internal preputial grafts (Mokhless et al. 2009). Grafts should be inlayed in the middle of the urethral plate during the TIP operation to eliminate exposing the area of the new urethral plate, promote re-epithelialization, and prevent scar formation. However, we could find only a few reports about this method. Asanuma et al. (2007) reported 28 cases of hypospadias who received the Snodgraft procedure, and preoperatively, the urethral meatus was coronal in two cases, distal shaft in seventeen cases, proximal shaft in six cases, and penoscrotal in three cases. Only one urethrocutaneous fistula developed in a patient (3.6 %), who required repair surgery 6 months after the urethroplasty. No patients had meatal stenosis, neourethral strictures, or urethral diverticula along the inlay graft. Shimotakahara et al. (2011) reported a random comparison between the Snodgraft and Snodgrass procedures in 50 cases, and the postoperative urethral opening/urethral stricture and urethral fistula following the Snodgraft procedure was lower than that following the TIP (*P* < 0.05 and *P* < 0.01, respectively). It has once again been shown that the Snodgraft procedure had significant advantages. The inlay buccal mucosa graft urethroplasty was also used in failed cases or complex urinary fistula repair and also produced satisfactory results (Hayes and Malone 1999; Ye et al. 2008). We found these results of interest. For patients, especially distal primary patients with longer urethral defects, mild or no chordee, and poor urethral plates, classic TIP should rarely be performed, and a buccal mucosa graft is unnecessary due to extra damage. An internal preputial graft should be considered to be complementary. Choosing the right method for hypospadias repair, achieving extremely good results for any primary situation, and investigating the efficacy and safety of the internal preputial graft was the focus of this study.

## Methods

Between January 2009 and December 2015, we performed a retrospective study consisting of approximately 508 cases of primary surgery with distal and moderate type hypospadias identified by the urethral meatus during surgery (Table 1). Patients with primary distal hypospadias who had mild or no chordee and good penile development were analyzed in this study. Patients were classified into the following 3 groups based on the procedures they received: (1) the TIP group (cases with good quality of plates); (2) inlay buccal mucosa graft; and (3) inlay buccal mucosa graft (cases with poor plates). The graft groups with poor urethral plates were randomly selected for one of the two procedures. Children from 6 months to 3 years of age (median 1.2 years) were included in this study. A total of 198 patients were treated with classic TIP urethroplasty, 150 patients were treated with inlay buccal mucosa graft urethroplasty, and 160 patients were treated with inlay internal preputial graft urethroplasty. All surgeries were performed by 2 senior pediatric urologists (Ye weijing^1^ and Liu yidong^2^). Written informed consent was obtained from the parents of all of the patients and control subjects before the initiation of the study. Ethical approval was obtained from the Institutional Review Board of Renji Hospital, which was affiliated with the Shanghai Jiao Tong University School of Medicine.

**Table 1:** The number of hypospadias identified by the urethral meatus during surgery

Urethral meatus		Surgical procedure			Total (n)
		TIP	BUCCAL	INLAY	
Corona	Count (n)	38	20	26	84
	% Within urethral meatus	45.2 %	23.8 %	31.0 %	
	% Within surgical procedure	19.2 %	13.3 %	16.3 %	
	% of Total	7.5 %	3.9 %	5.1 %	
Distal shaft	Count (n)	82	69	75	226
	% Within urethral meatus	36.3 %	30.5 %	33.2 %	
	% Within surgical procedure	41.4 %	46.0 %	46.9 %	
	% of Total	16.1 %	13.6 %	14.8 %	
Proximal shaft	Count (n)	78	61	59	198
	% Within urethral meatus	39.4 %	30.8 %	29.8 %	
	% Within surgical procedure	39.4 %	40.7 %	36.9 %	
	% of Total	15.4 %	12.0 %	11.6 %	
Total	Count (n)	198	150	160	508
	% Within urethral meatus	39.0 %	29.5 %	31.5 %	
	% of Total	39.0 %	29.5 %	31.5 %	

*TIP* classic snodgrass, *BUCCAL* inlay buccal mucosa graft, *INLAY* inlay internal preputial graft

### Classic TIP urethroplasty

A U-shaped skin incision was made surrounding the meatus, and two paramedian incisions were extended to the tip of the glans. We removed the unhealthy urethral tissue on the anterior wall and kept the urethral plate. If there was still chordee greater than 15 degrees, a dorsal tunica albuginea plication was conducted based on the methods of Baskin and Duckett (1994), in which an artificial erection was initiated. After that, an artificial erection was repeated until no chordee was identified. The incised urethral plate was tubularized without tension over a 6–8-Fr catheter (determined by the patients’ ages or the urethra) using non-interrupted 7–0 polyglactin sutures.

### Standards of the inlay internal preputial graft and inlay buccal mucosa graft urethroplasty

**Fig. 1:**
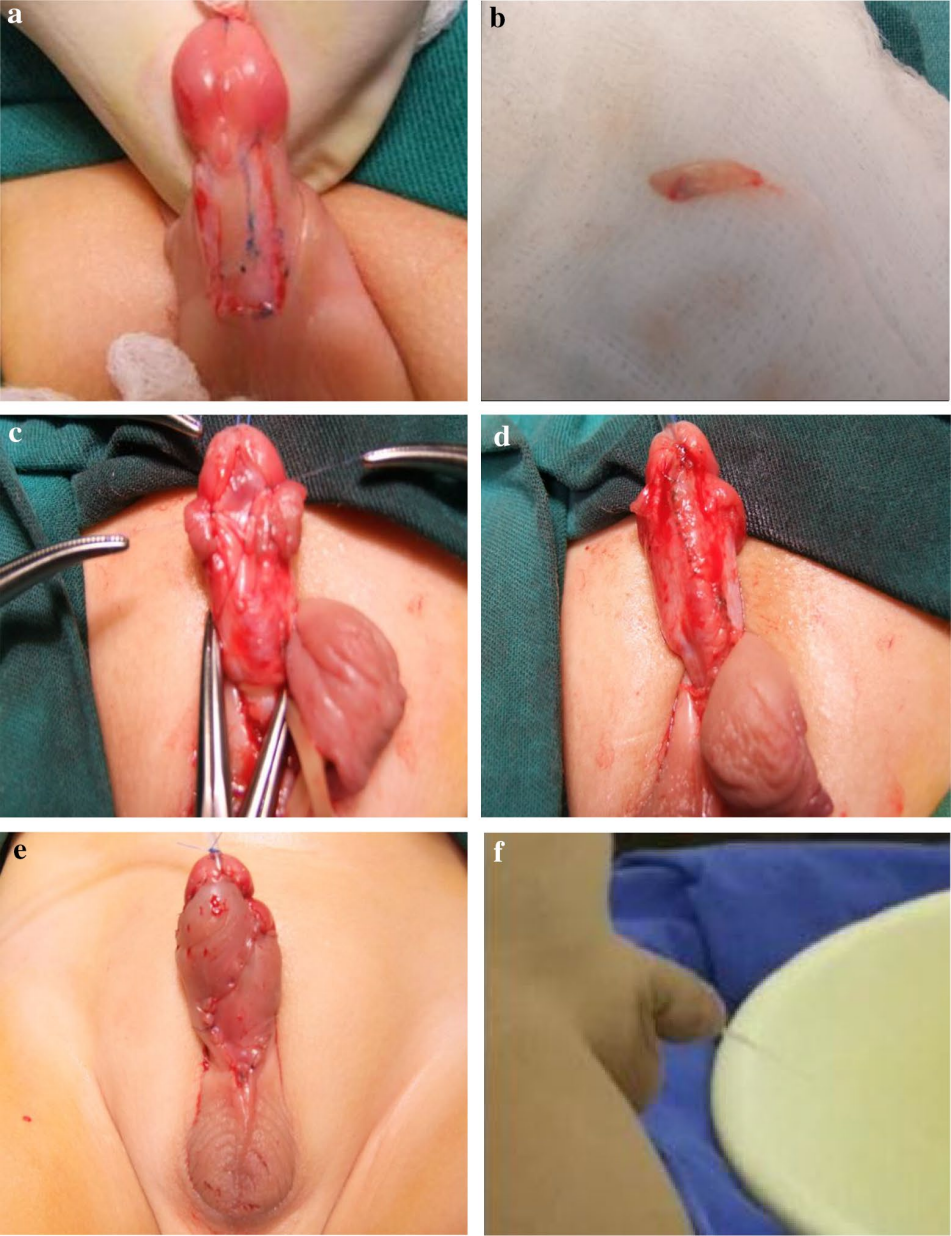
**a** The defect in the urethral plate upon opening; **b** harvesting of the internal preputial graft; **c** shaving the internal preputial graft; **d** suturing a ventral onlay flap to the urethral plate; **e** the cosmetic outcome; **f** the successful penile appearance

Because all of the patients could have underwent either surgery, we assigned the two procedures randomly. The main methods were as follows: measuring the width and length of the incised urethral plate, freeing the buccal mucosa graft or internal preputial graft, and suturing them into the urethral plate. The graft width was approximately 0.5‒0.8 cm and the length was approximately 0.5‒1.5 cm. Graft harvesting was performed by another surgeon working concurrently with the urethroplasty surgeon after determining the required graft length. Defattening of the graft was performed, and then, it was sutured as a ventral onlay flap to the urethral plate using 7/0 Maxon running subcuticular sutures. In all of the cases, a vascularized tunica vaginalis flap was used to cover the graft, and then, the glans wings were closed, followed by the skin cover. A compression dressing was applied for approximately 6 days (Fig. 1).

### Post-operative follow-up

The urethral catheter was removed after 2 weeks. The postoperative follow-up period was approximately 6‒24 months (mean: 18 months). For data analysis, our hospital used SPSS 13.0 software for statistical inspection. The rate comparison of urethral meatus and the outcome were performed by Chi square test, and *P* < 0.05 indicated that differences were statistically significant. We assessed the outcome of the cosmetic results by the HOSE score table [designed by Holland et al. (2001)]. The scores and the follow-up periods of the groups were analyzed by SPSS 13.0, and the comparison of rates was performed by *t* test with *P* < 0.05 indicating statistically significant differences. A graph analysis was performed using GraphPad 5.0.

A successful outcome was defined based on the presence or absence of complications, including fistula (which required additional intervention), diverticula (which could affect the urine stream and required surgery), meatal stenosis/stricture (with stricture apparent on a urethrogram requiring subsequent intervention), recurrent curvature greater than 30-degrees, glans dehiscence and/or skin reoperation with some bleeding, and a loss of stent (Keays et al. 2016).

## Results

**Fig. 2:**
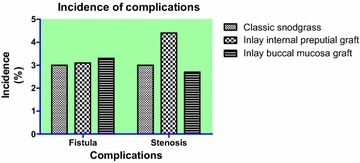
Retrospective complications and comparisons

**Fig. 3:**
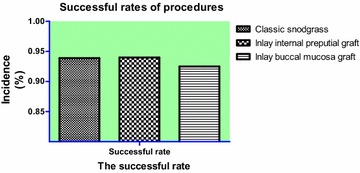
Successful rates of 3 procedures

**Fig. 4:**
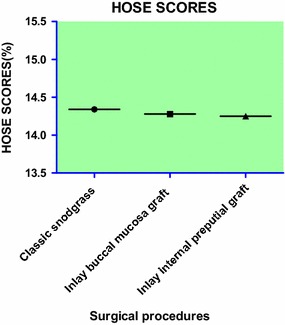
The comparisons of the different groups according to the HOSE table

Our results show that the incidence of urinary fistula was 3.0 % (6/198), and the incidence of meatal stenosis was 3.0 % (6/198) in the TIP group; the incidence of urinary fistula was 3.3 % (5/150), and the incidence of meatal stenosis was 2.7 % (4/150) in the inlay buccal mucosa graft group; and the incidence of urinary fistula was 3.1 % (5/160), the incidence of meatal stenosis was 4.4 % (7/160) in the inlay internal preputial graft group (Table 2; Fig. 2). No diverticula, recurrent curvature greater than 30-degrees, glans dehiscence or skin reoperation were observed in any of the patients. The success rates for each of the groups were 93.9 % (186/198), 94.0 % (141/150) and 92.5 % (148/160). All complications were found after 3 months. Compared to the TIP group, urethral meatus in the inlay buccal mucosa graft and the inlay internal preputial graft groups showed no statistically significant difference (Pearson, *P* > 0.05); the surgery success rate and complication rates showed no statistically significant differences (*P* > 0.05) (Fig. 3). The cosmetic outcomes (mean score) as rated by the HOSE table were 14.34, 14.28, and 14.25 for each respective procedure. The mean follow-up period of the three respective groups were 17, 18.5, and 18.5 months. There were no significant differences between the groups with respect to the cosmetic scores (*P* > 0.05, *P* = 0.47) (Fig. 4) and follow-up periods (*P* > 0.05, *P* = 0.23).

**Table 2:** Incidence of urinary fistula and meatal stenosis

Procedure group	n	Fistula (n/%)	Meatal stenosis (n/%)
Classic snodgrass	198	6/3.0	6/3.0
Inlay internal preputial graft	160	5/3.1	7/4.4
Inlay buccal mucosa graft	150	5/3.3	4/2.7

## Discussion

In 1994, Snodgrass reported (1994) a one-stage urethroplasty TIP for the distal and moderate types of hypospadias. He has successfully integrated the urethral plate incision with tubularization, producing a satisfying cosmesis. Then, a neomeatus was created. Tubularizing the urethral plate without tension should be emphasized in the case of strictures. In 2001, Lopes et al. (2001) reported that the new dorsal urethral plate area could regenerate through normal epithelium, which is called re-epithelialization. They had found that epithelial cells could move into the dorsal epithelial defect on postoperative day 2, and the re-epithelialization process was completed by postoperative day 5. Increased fibroblastic activity and early collagen deposition could be observed below the incised area on postoperative day 3. No further increased fibroblastic activity or excess collagen deposition appeared on postoperative day 21 (Lopes et al. 2001; Taneli et al. 2004). These studies promoted our current understanding of urethral healing after an incision. TIP has become increasingly popular because of its simplicity, versatility, and favorable cosmetic outcome; therefore, it has become the major choice for distal and moderate types of hypospadias.

With increasing surgical experience, TIP applications are also becoming more extensive and now include several types of hypospadias. However, newer applications introduced more complications, and several studies have indicated that the overall success rates decreased in patients having longer neourethras and poor urethral plates who have received classic TIP (Adayener and Akyol 2006; Braga et al. 2007; Perera et al. 2012; Asanuma et al. 2007).

Therefore, some scholars advocate that the graft should be sutured onto the urethral plate during the TIP surgery to eliminate exposing the area of the new urethral plate, stimulate re-epithelialization, and prevent scar formation. Since the inner preputial graft had been first described as an effective method for hypospadias repair with the advantage of reducing the risk of stenosis in 2000 (Kolon and Gonzales 2000), only a few studies of its modification have been reported using large sample sizes to prove the efficacy and safety of the Snodgraft procedure. Twenty-eight cases of hypospadias undergoing the Snodgraft procedure have been reported (Asanuma et al. 2007), and a urethrocutaneous fistula developed in only one patient (3.6 %). No patients had meatal stenosis, neourethral strictures, or urethral diverticula along the inlay graft. Shimotakahara et al. (2011) reported a random comparison between the Snodgraft and Snodgrass procedures in 50 cases. The postoperative urethral opening/urethral stricture and urethral fistula of the Snodgraft procedure was lower than those of the TIP (*P* < 0.05 and *P* < 0.01, respectively). It had been proven that the Snodgraft procedure has significant advantages. Acimovic M reported their overall success rate was 90.6 % on re-operative patients with anterior urethral strictures (Acimovic et al. 2016). Over the last decade, buccal mucosa gained popularity as the best substitute material for urethral reconstruction (Djordjevic 2014; Barbagli et al. 2005). Inlay buccal mucosa graft urethroplasty was also used in many other failed cases or complex urinary fistula repair but rarely in distal and moderate primary patients (Hayes and Malone 1999; Ye et al. 2008).

In our study, we performed a retrospective analysis on the three procedures using a large number of primary cases to make an accurate assessment of the outcomes. The success rates were 93.9 % (186/198), 94.0 % (141/150), and 92.5 % (149/160). Compared to the TIP group, the inlay buccal mucosa graft and the inlay internal preputial graft groups did not show any statistically significant differences in surgical success and complication rates (*P* > 0.05) (Fig. 3). The cosmetic outcomes (mean score) graded by the HOSE table were 14.34, 14.28, and 14.25. There was no significant difference among groups with respect to the cosmetic scores (*P* > 0.05, *P* = 0.47) (Fig. 4). Holland et al. (2001) had reported that a total score ≥ 14 would infer an acceptable outcome in the present era of hypospadias repair, with the proviso that the meatus is at least at the proximal meatus, with a single urinary stream and only moderate angulation of the penile shaft. So, we obtained an acceptable or satisfactory cosmetic result without significant differences between the groups based on the above-indicated scores. The inlay buccal mucosa graft and inlay internal preputial graft groups achieved satisfactory outcomes with TIP.

Buccal mucosa and internal preputial grafts share many advantages, such as a strong anti-infection ability, rich capillary beds, high survival rates, and abundant graft sources (Bapat et al. 2007; Holzle et al. 2012; Iselin and Webster 1998). Therefore, these grafts can be ideal candidates for urethral tissue in reconstruction. In addition, an internal preputial graft has the unique advantages of accessibility and low damage to the donor sites (Wilkinson et al. 2012). However, we advocate the inlay internal preputial graft for patients as part of our criteria instead of the buccal mucosa graft.

Based on the TIP, the internal preputial graft is obviously different from the oral mucosa. Therefore, except for the re-operative patients, an internal preputial graft could prevent ectopic trauma caused by obtaining oral mucosa, reduce anesthesia risks caused by ETGA, and save the cost of anesthesia. For the distal and moderate primary patients with longer urethral defects, narrow urethral plates, and mild or no chordee, the classic TIP should rarely be the first choice. Meanwhile, a buccal mucosa graft is unnecessary because of extra damage. Our study indicated that the internal preputial graft has the advantages of TIP and extended its applications.

It should be noted that the inlay internal preputial graft that Snodgrass applies to patients for the first time was only applied at the distal and moderate types. We summarized the following two groups who are no longer suitable for this procedure: (1) For the re-operative cases, the internal preputial graft and the original urethral plate have been commonly damaged; therefore, it is hard to obtain a satisfying outcome. (2) For patients who need to transect the poor urethral plates with width less than 5 mm (measured in operation), bad quality and serious fibrosis, the internal preputial graft cannot fully play the role of re-epithelialization on the transected plate. The graft is only used as a patch for the hypospadias urethra instead of tubing into the urethra. The inlay internal preputial graft Snodgrass is rarely preferred for these groups.

Our study has many limitations as follows: (1) Patients selection as we only selected patients with distal and moderate primary hypospadias, good penile development, and mild or no curvature and (2) a short follow-up period. A short follow-up period may exert a great effect on the complication rates (Spinoit et al. 2013). We have a shorter period follow up than some other reports.

In general, the Snodgrass inlay internal preputial graft could be used for patients with normal penile development, mild or no chordee, poor urethral plate development and distal or moderate types of hypospadias except for the re-operative patients, which could be used to avoid the damage of harvesting the donor area of the oral mucosa and produce the advantages of the classic snodgrass procedure. The Snodgrass inlay internal preputial graft has a high success rate, favorable cosmetic outcomes, and no extra damage. Because our study has a short follow-up period, we will investigate a longer outcome in our next study.

## Conclusion

As the Snodgraft inner foreskin does not appear to be worse than the buccal mucosa graft, this may be a good method for hypospadias repair, and this method is not inferior to TIP.

## Abbreviations

TIP: Classic snodgrass
BUCCAL: Inlay buccal mucosa graft
INLAY: Inlay internal preputial graft
ETGA: Endotracheal tube intubation general anesthesia
HOSE table: Hypospadias objective scoring evaluation
